# Primary Low-Grade Astrocytoma of the Spine With Secondary Cerebral Metastasis: A Case Report and Comprehensive Review of the Literature

**DOI:** 10.7759/cureus.10030

**Published:** 2020-08-25

**Authors:** Jason D Vadhan, Daniel G Eichberg, Long Di, Glen Manzano, Michael Ivan, Ricardo J Komotar

**Affiliations:** 1 Osteopathic Medicine, Nova Southeastern University, Miami, USA; 2 Neurological Surgery, University of Miami Miller School of Medicine, Miami, USA

**Keywords:** spine, brain tumor, neurosurgery, metastasis, astrocytoma, glioma

## Abstract

An astrocytoma is a subclassification of glioma, with primary spinal manifestations accounting for less than 10% of all spinal cord tumors, with the majority encompassing low-grade features. It is even more uncommon for such lesions to demonstrate intracerebral metastasis. We report such an occurrence in a 39-year-old female who initially presented with an intramedullary and intradural mass from T10-L1, as well as secondary metastasis to the mesial right temporal lobe and cerebellum upon clinical follow-up. Surgical resection of the spine and subsequent temporal lobe biopsy confirmed high-grade glioma. Given the rarity and poor prognosis of spinal gliomas with cerebral metastasis, we also summarize all previously reported cases to date. We recommend that physicians maintain an index of suspicion for spinal gliomas in young patients with cord compression related symptoms outside the event of traumatic injury.

## Introduction

Primary spinal cord tumors are uncommon, constituting approximately 2-10% of all Central Nervous System (CNS) tumors, with the overwhelming majority (70%) of such lesions being of low-malignant potential [1]. The occurrence of spinal cord gliomas is even less common, arising in approximately 0.22 per 100,000 individuals [2]. Such tumors have been historically shown to arise within the cervical region in primary cases and typically occur in younger male patients (< 30 years old) [3]. Despite the best treatment with surgery and adjuvant therapy, overall survival remains poor.

We present the case of a patient with a low-grade astrocytoma of the thoracic spinal cord who then developed additional cervical and thoracic spread, followed by high-grade transformation upon cerebral metastasis. We discuss the pertinent clinical, operative, and histopathological findings. We also performed a comprehensive review of the literature of all reported cases of primary spinal astrocytomas with brain parenchyma extension.

## Case presentation

A 38-year-old female presented with a three-month history of progressive low back pain radiating to bilateral lower extremities with paraparesis and radiating shock-like pain with Valsalva. In addition, she reported gait instability, falling episodes, and bilateral foot drop (left worse than right) with muscle laxity. She denied any bowel or bladder incontinence. Physical exam demonstrated gait instability, impaired toe walking, impaired heel walking, impaired tandem gait, and an antalgic gait. The bilateral upper extremity strength was 5/5. Lower extremity exam demonstrated 4/5 psoas strength bilaterally, 3/5 quadriceps strength bilaterally, 2/5 (left), and 1/5 (right) tibialis anterior strength, 2/5 (left) 1/5 (right) extensor hallucis longus, 3/5 gastrocnemius strength bilaterally. All upper extremity deep tendon reflexes were 2/4 bilaterally. Patellar and ankle reflexes were 4/4 bilaterally. There were no changes in sensation among upper and lower extremities bilaterally. Babinski and Hoffman signs were absent. Finger to nose coordination was unremarkable.

**Figure 1:**
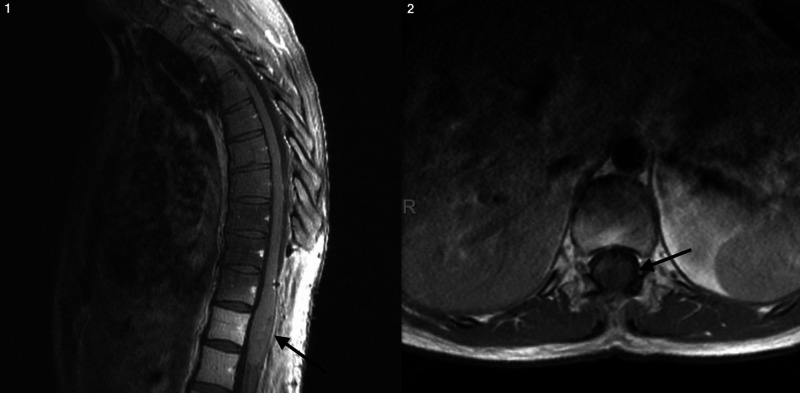
Sagittal (1) and axial (2) T1 MRI with contrast demonstrating an intramedullary tumor (arrow) that was found to be a WHO Grade II astrocytoma Abbreviations: S, superior; I, inferior; L, left; R, right; A, anterior; WHO, World Health Organization

Magnetic resonance imaging (MRI) demonstrated a large intradural intramedullary mass extending from T10-L1 (Figure 1). The lesion was approximately 1.7 x 2.0 x 7.6 cm in size. Cranial imaging was negative for any lesions.

The patient underwent surgical resection, by which baseline motor evoked potentials (MEP) were recorded. Baseline MEPs were abnormal, consistent with the preoperative presence of paraparesis on admission. A vertical midline incision was made overlying the thoracolumbar junction, and a complete laminectomy from T10-L1 was performed. The spinal cord was abnormal, demonstrating an enlarged component, consistent with an intramedullary tumor. The surface of the spinal cord appeared discolored, and a midline myelotomy was performed. A large, firm, rubbery, pale mass that encompassed the overwhelming majority of the spinal cord was encountered, internally debunked, and sent for frozen pathology, which confirmed a World Health Organization (WHO) grade II diffuse astrocytoma. The tumor demonstrated extensive spread, and several areas were encountered that did not demonstrate a definable plane. The myelotomy was extended from the bottom of T10 down to L1. Serial MEPs were performed throughout the bulking process, which eventually indicated the loss of distal right lower extremity MEP and possible left lower extremity MEP loss. Given the clear intraoperative findings that this invasive tumor was without clear margins, and the evidence of MEP change, the surgical resection remained as a subtotal resection. The wound was then irrigated with copious amounts of antibiotic irrigation and hemostasis obtained. The dura was then closed in a watertight fashion and copiously irrigated with 3 liters of pulsatile antibiotic irrigation. During the closure of the case, there was an improvement in the left lower extremity MEPs and a questionable slight improvement in the right distal lower extremity MEPs. Cerebrospinal fluid (CSF) cytology was negative.

Following surgical resection, physical exam demonstrated 5/5 upper extremity muscle strength bilaterally. Lower extremity physical exam demonstrated 5/5 (left) and 4/5 (right) psoas strength, 5/5 (left) 3/5 (right) quadriceps strength, 0/5 dorsiflexion strength bilaterally, 1/5 (left) and 0/5 (right) extensor hallucis longus strength, and 0/5 (left) and 4/5 (right) gastrocnemius muscle strength. New-onset paresthesia was reported from L4 to distal right lower extremity, as well as from L1 to distal left lower extremity. 

**Figure 2:**
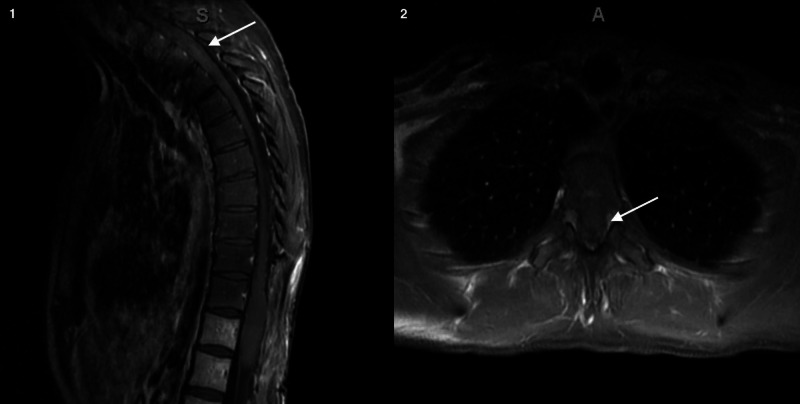
Sagittal (1) and axial (2) T1 MRI with contrast of T1-T5 demonstrating a new expansile cord lesion with heterogeneous signal within the thoracic spinal cord Abbreviations: S, Superior; I, inferior; L, Left; R, Right; A, Anterior.

**Figure 3:**
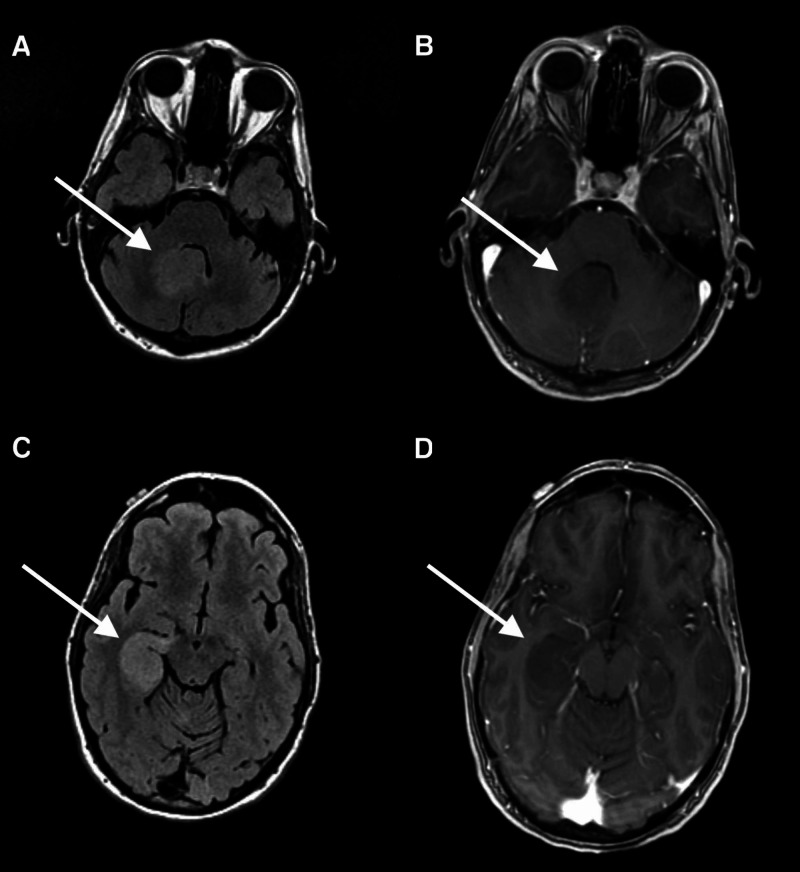
Axial FLAIR and T1 MRI with contrast demonstrating non-enhancing FLAIR hyperintense masses in the right cerebellum (Image A, B) and mesial right temporal lobe (Image C, D) compatible with multifocal Grade III astrocytoma FLAIR: fluid attenuated inversion recovery

Due to social considerations, the patient began a seven-week course of chemoradiation two months postoperatively. This included six cycles of adjuvant temozolomide. Over the next two months, the patient reported progressive lower extremity weakness, dizziness, fatigue, poor appetite, and depressed mood. Repeat MRI of the spine revealed a new expansile cord lesion with heterogeneous T2 signal within the thoracic spinal cord spanning from T1 to T5 (Figure 2). She was subsequently admitted and began intravenous dexamethasone. She also underwent brain MRI that revealed non-enhancing T2 Fluid-attenuated inversion recovery (FLAIR) hyperintense mass located in the mesial right temporal lobe, with diffuse involvement of the hippocampus, measuring 1.6 x 5.1 x 2.4 cm, with diffuse subependymoma involvement and compression of the temporal horn (Figure 3). In addition, another area of non-enhancing FLAIR hyperintense signal located in the vermis of the cerebellum measuring 2.6 x 2.9 x 2 cm with compression of the anterior aspect of the fourth ventricle without evidence of hydrocephalus.

**Figure 4:**
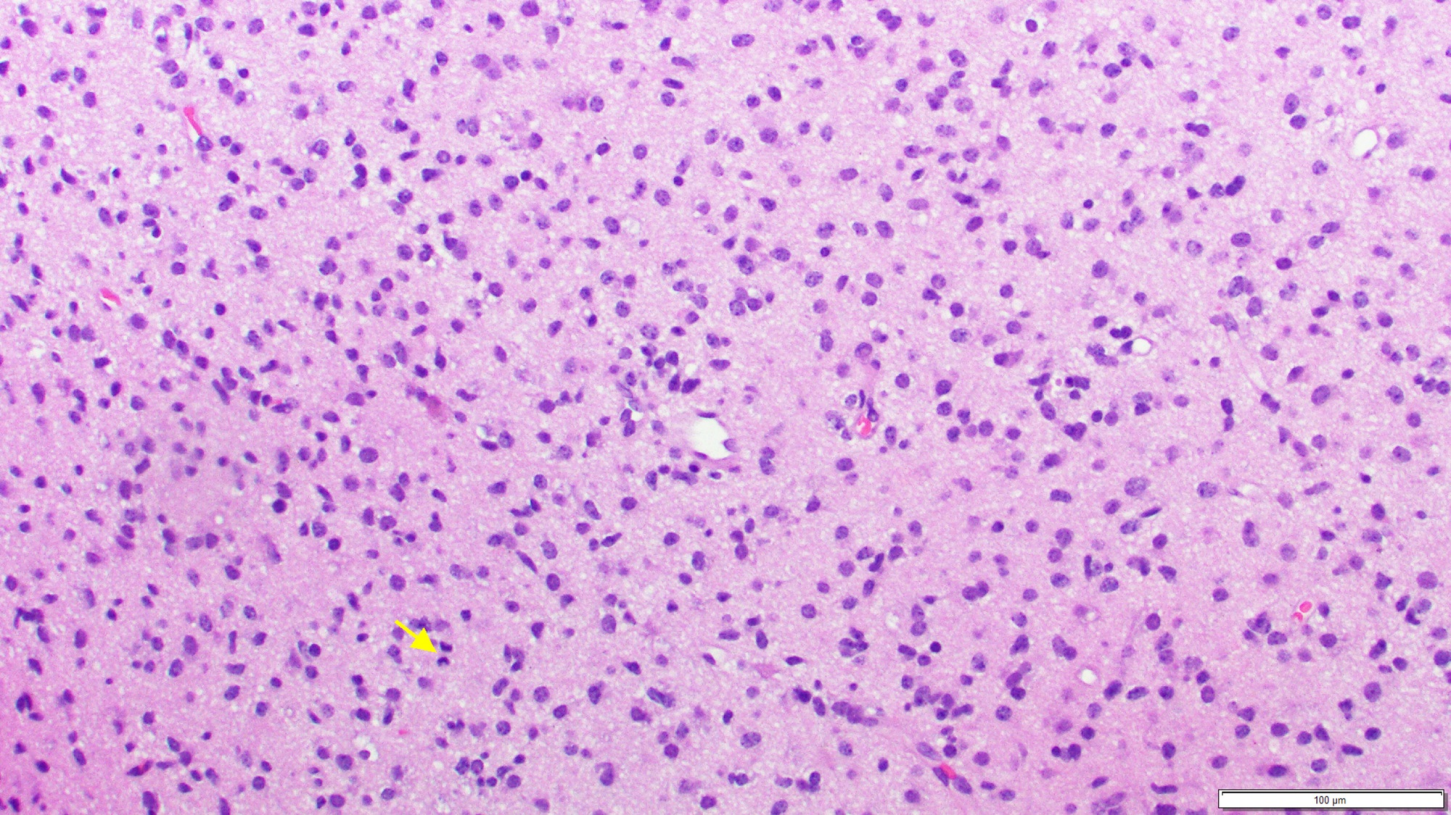
Right temporal lobe biopsy demonstrating WHO grade III anaplastic astrocytoma consisting of infiltrating hyperchromatic nuclei and numerous mitotic figures (yellow arrow)

A stereotactic needle biopsy of the right temporal lobe was performed, with subsequent histopathology demonstrating WHO grade III anaplastic astrocytoma without microvascular proliferation or necrosis (Figure 4). Moderately cellular astrocytic proliferation with two to three mitotic figures detected on hematoxylin and eosin stain was demonstrated. Immunohistochemical studies were positive for glial fibrillary acidic protein (GFAP) and oligodendrocyte transcription factor 2 (OLIG2), with P53 demonstrating <20% labeling. IDH-1R132H labeling was equivocal, and ATRX expression was retained. Phosphohistone H3 (PHH3) demonstrated mildly increased mitotic figures. Ki67 labeling index was 5%. H3K27M mutation was noted.

The patient survived for only eight months post-diagnosis.

Literature review

The PubMed database and all major neurosurgery journals were searched during June of 2020 using the keywords “glioma”, “astrocytoma”, "glioblastoma", “spine”, and “cerebral extension”, alone or in combination to obtain articles fitting the inclusion and exclusion criteria. The inclusion criteria were high-grade astrocytomas or glioblastomas involving the spine with secondary brain parenchyma metastasis.

To date, 96 cases of primary spinal gliomas with secondary intraparenchymal manifestation have been reported since 1908, including this case (Table 1). These lesions occurred in patients ranging from six months to 65 years old, with the majority presenting under the age of 40 years (Mean: 20, 95% CI: 17-23.28 months). Among such reported cases, the long-term survival and outcome remain poor with a mean average survival of 24 months from diagnosis (95% CI: 13.07-33.99 months). There appears to be a slight predilection for this subtype among men over females (1.46:1).

**Table 1:** Summary of previous reported cases of primary spinal gliomas with secondary intracranial metastasis Abbreviations: F, female; M, male; C, cervical spine; T, thoracic spine.

Author	Age	Sex	Primary Tumor Site	Brain Metastasis	Survival since presentation	Histology
Abel et al., 2006 [4]	4	M	T7-T9	cerebral cortex	Not specified	III
Allen et al., 1998 [5]	4	M	Corticomedullary	Leptomeninges	14 months	III
Allen et al., 1998 [5]	13	F	Cervical Spine	Leptomeninges	15 months	IV
Allen et al., 1998 [5]	12	F	Thoracic Spine	Leptomeninges	20 months	III
Allen et al., 1998 [5]	15	M	Cervical spine	Leptomeninges	8 months	IV
Amlashi et al., 2006 [6]	6	F	T7-T9	cerebral cortex	4 years	II
Ando et al., 2010 [7]	65	F	C1-C4	Pons	7 weeks	IV
Andrews et al., 1978 [4]	45	M	T12, Conus medullaris	Septal region, right lateral ventricle, right cerebellum and septum pellucidum	13 months	IV
Asano et al., 1990 [4]	23	F	T11-L1	4th ventricle, anterior horn of the left lateral ventricle, septum pellucidum and pituitary gland	12 months	IV
Battaglia et al., 2007 [5]	11	M	T4-T5	Leptomeninges, hippocampus	6 months	IV
Bell et al., 1988 [4]	2	M	C3-C7	Basal Cistern	17 months	II
Bell et al., 1988 [4]	3	M	C2-C7	Interpeduncular cistern	Not specified	II
Bonde et al., 2007 [5]	16	M	conus medullaris	Cervicomedullary junction, pituitary stalk	13 months	IV
Caroli et al., 2005 [5]	6	M	T9-T11	Frontal lobe	13 months	IV
Chida et al., 1995 [8]	22	M	Cervical spine	Brainstem and cerebellum	3 months	IV
Ciappetta et al., 1991 [9]	59	M	C3-C7	Left occipital lobe	29 months	IV
Civitello et al., 1988 [6]	3	N/A	Cervical spine	Basal Cistern, tentorium, chiasm, hypothalamus	Not specified	II
Civitello et al., 1988 [6]	6	N/A	Cervical spine	cerebral white matter, vermis	Not specified	II
Claus et al., 1995 [4]	43	M	Conus medullaris	Brainstem, cerebellum, septum pellucidum, ventricles	5 years	II (progression to IV)
Cohen et al., 1988 [4]	17	F	Thoracic spine	Subarachnoid space and brainstem	10 months (post-op)	IV
Cohen et al., 1989 [4]	9	M	Cervical spine	Brainstem	1 month (post-op)	IV
Cohen et al., 1989 [4]	14	M	Conus medullaris	Septum pellucidum	4 months (post-op)	IV
Cohen et al., 1989 [4]	10	F	Cervical spine	Subarachnoid space	5 months (post-op)	IV
Cohen et al., 1989 [4]	16	F	Conus medullaris	Septum pellucidum	6 months (post-op)	IV
Cursiefen et al., 1998 [8]	16	M	C5-T1	Supratentorial	5 months	IV
Demir et al., 2010 [6]	8	F	T8-T9	cerebellum	Not specified	II
Derinkuyu et al., 2015 [5]	9	F	T8-T10	brainstem	8 months	IV
Eade et al., 1971 [4]	21	F	thoracic spine	Subarachnoid space, ventricles	11 months	II
Eade et al., 1971 [4]	19	M	Conus medullaris	Subarachnoid space, ventricles	6 months	II
Eade et al., 1971 [4]	21	F	thoracic spine	Subarachnoid space, ventricles	8 months	II
Eden, 1938 [4]	11	M	T4-T5	Cerebellar leptomeninges, perimedulla and hippocampus	7 months	IV
Elsamaloty et al., 2006 [10]	20	M	Conus medullaris	Cervicomedulary junction, suprasellar cistern, left lateral ventricle and right cerebellum	13 months	IV
Galarza et al., 2006 [6]	0.5	M	C1-C6	pons	Not specified	I
Galarza et al., 2006 [6]	2	M	T8-T9	cerebellum	Not specified	I-II
Greenfield et al., 1934 [4]	48	M	Cauda Equina	Subarachnoid Space, ventricles	6 years	Meduloepithelioma?
Hely et al., 1985 [4]	19	F	Conus medullaris	Subarachnoid space, ventricles, thalamus, hypothalamus, midbrain, pineal gland	28 months	II
Hely et al., 1985 [4]	38	F	T2-T3	Subarachnoid space, ventricles, hypothalamus, brainstem, thalamus	9 months	III
Hukin et al., 2003 [6]	5	N/A	Not specified	Not specified	5 months	II
Hukin et al., 2003 [6]	8.7	N/A	Not specified	Not specified	60 months	ganglioglioma
Inagawa et al., 1995 [4]	16	M	Cervical Spine	Medulla	7 months	II
Jeong et al., 2010 [4]	22	M	T3-T11	Lateral ventricles, septum pellucidum	Not specified	III
Johnson et al., 1987 [4]	9	F	T11-L3	Subarachnoid space, ventricles	17 months	III
Kataria et al., 2011 [8]	15	F	T11-L1	brainstem	3 months	III
Kawanishi et al., 1993 [8]	50	M	T11-T12	Cerebellum, cingulate gyrus and sylvian fissure	18+ months	IV
Kawashima et al., 2004 [5]	8	F	C7-T11	Cerebellum, brainstem	12 months	IV
Kendrick et al., 1987 [4]	41	F	Thoracic spine	Subarachnoid space	Not specified	IV
Kim et al., 2011 [5]	16	F	T12-L1	Not specified	12 months	IV
Klase et al., 2007 [4]	1.5	F	Cervicothoracic spine	Cerebellum	18 years	II (progressed to IV)
Klepstad et al., 2001 [5]	12	F	Cervical spine	Brainstem, medulla	2 months	IV
Kokkalis et al., 2016 [5]	12	M	T4-T8	Bilateral frontal midline lesion and corpus callosum	20 months	IV
Kopelson et al., 1982 [11]	32	F	Not specified	midbrain	Not specified	II
Kumar et al., 2019 [5]	4	M	Cervicothoracic spine	Brain stem, cerebellum and tuber cinereum	4 months	IV
Linsenmann et al., 2015 [8]	35	M	T2-T3	Left frontal lobe	19 months	IV
Mallory et al., 1908 [4]	N/A	N/A	Lumbar Spine	Subarachnoid Space, cervical cord, pons and cerebellum	Not specified	III-IV
Medhkour et al., 2005 [8]	20	M	T12-L1	Thoracic and cervical spine, medulla, pontomedullary junction, cerebellum, suprasellar cistern and left lateral ventricle	11 months	IV
Morais et al., 2012 [12]	19	M	T6-T11	Pituitary stalk, inter-peduncular cistern and left superior cerebellar peduncle	21 months	IV
Mori et al., 2012 [5]	10	F	Holocord	Corticomedullary junction and pituitary stalk	14 months	IV
Ng et al., 2001 [4]	9	F	C5-C7	Sylvian Fissures, brain stem and cerebella sulci	Not specified	I
Nunn et al., 2017 [13]	31	M	conus medullaris	leptomeninges	14 months	IV
O'Connell et al, 1946 [4]	16	M	T6-T12	Pontine, interpeduncular cistern and inferior cerebral hemispheres	16 months	IV
Ozgiray et al., 2013 [8]	54	F	C3-C4	Medullary-pontine junction, cerebellum, suprasellar cistern, left lateral ventricle	2 months	IV
Peraud et al., 2004 [4]	14	M	T11-T12	Ventricles, frontal lobe	Not specified	II-III
Perese et al., 1959 [4]	39	M	Conus medullaris	Subarachnoid space, ventricles, cerebellum	28 months	I - II
Perilongo et al., 2002 [8]	7	M	C5-C6	cerebellum, cerebrum, brain stem	3 months	II
Perilongo et al., 2002 [8]	3	F	C7-T5	cerebellum, brain stem, temporal lobes	9 years	II
Perilongo et al., 2002 [8]	12	M	C7-T1	cerebellum, occipital love, lateral ventricles	Not specified	II
Purkayastha et al., 2018 [14]	23	M	T8-T10	Frontal, occipital horn and septum pellucidum	8 months	IV
Rubenstein et al., 1970 [11]	17	F	filum terminale	ventricles	29 years	ependymoma
Ruppert et al., 2010 [4]	54	F	T7-T10, cervical and lumbar spine	Sylvian fissure, suprasellar cistern and posterior fossa	Not specified	III
Russell et al., 1949 [4]	37	M	Cervical spine	Subarachnoid space, ventricles	5 months	Oligodendroglioma
Russell et al., 1959 [4]	11	F	Cervical spine	Subarachnoid space, ventricles	6 months	IV
Russell et al., 1971 [4]	16	F	Conus medullaris	Subarachnoid space	Not specified	III-IV
Salazar et al., 1976 [4]	N/A	N/A	Not specified	Not specified	Not specified	IV
Saleh et al., 1987 [15]	9	M	T6-T8	Third ventricle	6 months	II
Sanei-Sistani et al., 2020 [16]	6	M	T8-T12	posterior fossa, left lateral ventricle, cerebellopontine angle, left meckel cave	28 months	II
Santi et al., 2003 [17]	3	M	T7	not specified	13 months	IV
Santi et al., 2003 [17]	18	M	T10-T12	not specified	14 months	III-IV
Santi et al., 2003 [17]	27	M	T12-L2	cerebellum	16 months	IV
Santi et al., 2003 [17]	20	M	T11-L1	Optic Nerve, brain base	3 months	III-IV
Santi et al., 2003 [17]	45	M	C1-C7	brainstem, infundibulum, and cranial nerve roots	3 months	III-IV
Santi et al., 2003 [17]	29	M	Cervical spine	subarachnoid space	42 months	IV
Santi et al., 2003 [17]	22	F	T12	Supratentorial	6 months	III-IV
Sarabia et al., 1986 [4]	54	M	thoracic spine	Subarachnoid space, ventricles, corpus collosum, optic chiasm	13 months	III
Schlereth et al., 2012 [4]	63	M	T6-T7	Temporal lobe	5 weeks	III-IV
Simonati et al., 1978 [4]	19	F	Not specified	Subarachnoid space, ventricles	5 years	II
Song et al., 2020 [5]	7	F	T2-T5	cerebellum, pons, ventricles, hippocampus, basal ganglia, paraventricles, frontal lobe, temporal lobe, pineal gland, thalamus, cerebral peduncle	1 month	IV
Stecco et al., 2005 [5]	14	M	T12-L1, Conus medullaris	Posterior fossa	9+ months	IV
Strik et al., 2000 [8]	31	F	T10-T11	Medulla, cerebellum and suprasellar region	15 months	IV
Sun et al., 2009 [5]	14	M	Conus medullaris	Lateral ventricle	16 months	IV
Takara et al., 1985 [8]	20	M	T5-T8	subarachnoid space, ventricles, cerebellum, brainstem	5 months	IV
Tashiro et al. 1976 [4]	12	F	Conus medullaris	Cerebellum, hypothalamus, brainstem and thalamus	11 months	IV
Umezu et al., 1992 [4]	40	M	C2-C4	Leptomeninges, ventricles, basal cistern and prepontine cistern	14 months	III
Vassilyadi et al., 2005 [6]	3	M	T12-L1	brainstem, suprasellar region, quadrigeminal system and around the middle cerebral artery	Not specified	II-III
Yamagami et al., 1990 [11]	44	M	Conus medullaris	Subarachnoid space, ventricles, frontal lobe, basal ganglia	7 years 8 months	II-III
Yamashita et al., 2001 [4]	43	F	T7-T9	Brainstem, cerebellum, cerebral cortex	2 years	III
Yan et al., 2017 [5]	10	M	T11-L1, Conus medullaris	Left apical lobe, right cerebellar vermis, corpus callosum, basal ganglia and lateral ventricle	14 months	IV
Vadhan et al., 2020 (current case)	39	F	T10- L1	Mesial right temporal lobe, hippocampus and subependymoma	8 months	II-III

The most common location for tumor involvement was the thoracic spine, which was in agreement in a previous survey published by Linsenmann et al. [8]. Varying levels of resections were attempted (both within the spine and brain), demonstrating no remarkable differences in outcomes.

## Discussion

Tumors of the spinal cord can either be primary or (more commonly) of metastatic origin. Primary spinal tumors are most often intramedullary and are rare even within the realm of CNS neoplasms, accounting for less than 10% of all primary CNS lesions [18]. Among the intramedullary spinal neoplasms, astrocytomas and ependymomas make up the majority of cases, with gliomas accounting for less than 0.22 per 100,000 [3].

While the isolated occurrence of these tumors is certainly uncommon, subsequent metastasis of these lesions into the intraparenchymal space is even rarer.

A striking feature of our particular case was the supratentorial dissemination of the spinal lesion, which gave a progressive and multifocal picture. Of note, although it is certainly possible that the brain metastases are due to CSF dissemination and seeding during the initial surgery, it is also possible that the tumor disseminated postoperatively, given the aggressiveness and invasiveness seen with the H3K27M mutation profile.

The H3K27M mutation is an important consideration regarding high-grade gliomas, as the prognosis of H3K27M gliomas remains poor, and even less favorable than Glioblastoma Multiforme (GBM), with few options in treatment. One clinical trial worth noting has demonstrated efficacy and an exceptional safety profile targeting gliomas with such mutations via a selective dopamine receptor D2/3 antagonist (ONC201), regardless of age or tumor location [19]. 

**Figure 5:**
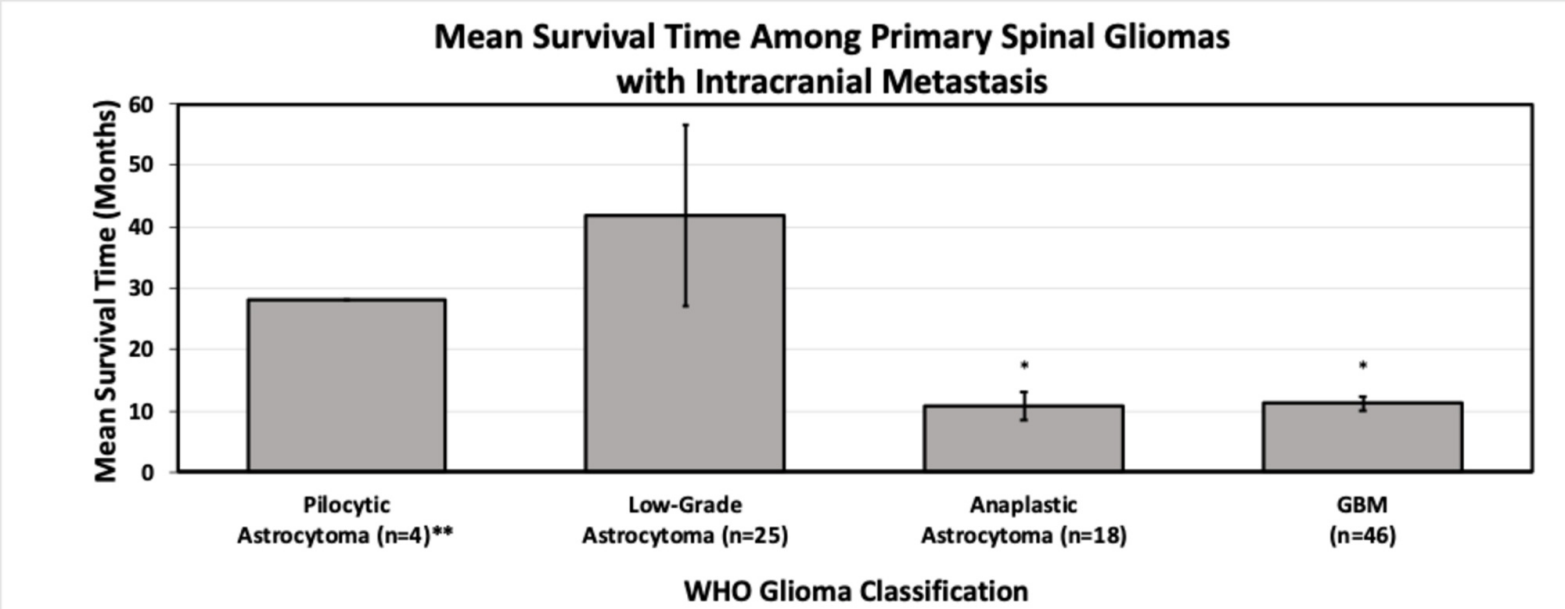
Mean survival time among cases of primary spinal gliomas with intracranial metastasis Cases with advanced disease were classified according to the initial grade discovered. *Anaplastic astrocytoma and GBM demonstrated a reduction in mean survival time relative to low-grade astrocytomas and pilocytic astrocytomas **Among the four reported cases of pilocytic astrocytomas, only one study reported a mortality

Our assessment highlights several patterns regarding the presentation and treatment of these tumors. First, primary spinal high-grade gliomas most commonly occur in young males (1.46:1 male: female) with a mean age of 20 years old (95% CI 17.0 to 23.28). This is in stark contrast from primary intracranial gliomas, which have a mean age of diagnosis of 60.4, as well as the overall incidence of primary spinal cord tumors overall [19]. Second, and unsurprisingly, the typical presenting symptoms for patients with spinal astrocytomas and cerebral metastasis are the result of cord compression (including pain, paresthesia, weakness, gait imbalance, and incontinence). Third, with regards to treatment, surgical resection remains the mainstay of treatment. However, despite such measures, survival outcomes remain poor, with increasing stage correlated with decreased mean survival time (Figure 5). Interestingly, high-grade gliomas demonstrated a similarly poor outcome relative to GBM. Both anaplastic astrocytomas and GBMs demonstrated reduced mean survival time relative to low-grade astrocytomas when using an analysis of variance (p < 0.05).

In sum, given the consistent constellation of symptoms portraying cord compression, and the unique age of presentation of high-grade spinal gliomas, we recommend providers maintain an index of suspicion for such patient presentations in practice when instances of traumatic injury as a potential cause are not apparent. Lastly, because secondary cranial metastasis is possible (although admittedly uncommon) we recommend routine brain imaging surveillance of all patients with primary high-grade gliomas.

## Conclusions

Primary high-grade gliomas of the spine with cerebral metastasis is a rare occurrence with a poor prognosis and most commonly presents in a unique patient population. Although surgical resection is commonly attempted, tumor recurrence and three-year mortality still remain close to 100%. Following our evaluation of the data, we recommend that providers maintain an elevated index of suspicion for spinal high-grade gliomas in young persons who present with cord compression related symptoms outside the realm of traumatic injury and that all patients with such tumors undergo aggressive surveillance imaging to monitor for secondary cranial spread.

## References

[1] Engelhard HH, Villano J, Porter KR (2020). Clinical presentation, histology, and treatment in 430 patients with primary tumors of the spinal cord, spinal meninges, or cauda equina. J Neurosurg Spine.

[2] Hsu S, Quattrone M, Ostrom Q (2011). Incidence patterns for primary malignant spinal cord gliomas: a surveillance, epidemiology, and end results study. J Neurosurg Spine.

[3] Ando K, Matsuyama Y, Sakai Y (2010). Cervical intramedullary glioblastoma with intracranial dissemination: description of a rapidly progressing case and a literature review. J Musculoskel Res.

[4] Schlereth T, Nguyen-Huu BK, Müller H (2012). Intracranial spreading of a spinal anaplastic astrocytoma. J Neurol.

[5] Song D, Xu D, Gao Q, Hu P, Guo F (2020). Intracranial metastases originating from pediatric primary spinal cord glioblastoma multiforme: a case report and literature review. Front Oncol.

[6] Demir HA, Varan A, Akyüz C (2011). Spinal low-grade neoplasm with leptomeningeal dissemination mimicking tuberculous meningitis in a child. Childs Nerv Syst.

[7] Ando K, Matsuyama Y, Sakai Y. (2010). Cervical intramedullary glioblastoma with intracranial dissemination: description of a rapidly progressing case and a literature review. J Musculoskel Res.

[8] Linsenmann T, Westermaier T, Vince GH (2015). Primary spinal glioblastoma multiforme with secondary manifestation as a cerebral “angioglioma.” Literature review and case report. J Neurol Surg Rep.

[9] Ciappetta P, Salvati M, Capoccia G, Artico M, Raco A, Fortuna A (1991). Spinal glioblastomas: report of seven cases and review of the literature. Neurosurgery.

[10] Elsamaloty H, Zenooz NA, Mossa-Basha M (2006). Glioblastoma multiforme (GBM) of the conus medullaris with brain and brain stem metastases. Eur J Radiol.

[11] Yamagami T, Kikuchi H, Higashi K, Goto Y, Imataka K (1990). Intracranial metastasis of a spinal cord astrocytoma--case report. Neurol Med Chir (Tokyo).

[12] Morais N, Mascarenhas L, Soares-Fernandes JP, Silva A, Magalhães Z, Moreira Da Costa J (2013). Primary spinal glioblastoma: a case report and review of the literature. Oncol Lett.

[13] Nunn A, Polyzoidis S, Piechowski-Jozwiak B, Brazil L, Ashkan K (2017). Primary glioblastoma multiforme of the conus medullaris with leptomeningeal metastasis. J Neurol Sci.

[14] Purkayastha A, Sharma N, Sridhar MS, Abhishek D (2018). Intramedullary glioblastoma multiforme of spine with intracranial supratentorial metastasis: progressive disease with a multifocal picture. Asian J Neurosurg.

[15] Saleh J, Afshar F (1987). Spinal cord astrocytoma with intracranial spread: detection by magnetic resonance imaging. Br J Neurosurg.

[16] Sanei-Sistani S, Miri-Aliabad G, Dahmardeh H, Montazeran M, Jahantigh M, Zare M (2020). Intracranial metastases of intramedullary spinal cord low-grade astrocytoma. Indian J Med Paediatr Oncol.

[17] Santi M, Mena H, Wong K, Koeller K, Olsen C, Rushing EJ (2003). Spinal cord malignant astrocytomas. Clinicopathologic features in 36 cases. Cancer.

[18] Chi AS, Tarapore RS, Hall MD (2019). Pediatric and adult H3 K27M-mutant diffuse midline glioma treated with the selective DRD2 antagonist ONC201. J Neurooncol.

[19] Pan IW, Ferguson SD, Lam S (2015). Patient and treatment factors associated with survival among adult glioblastoma patients: a USA population-based study from 2000-2010. J Clin Neurosci.

